# Entropy and bispectral index for assessment of sedation, analgesia and the effects of unpleasant stimuli in critically ill patients: an observational study

**DOI:** 10.1186/cc7015

**Published:** 2008-09-16

**Authors:** Matthias Haenggi, Heidi Ypparila-Wolters, Christine Bieri, Carola Steiner, Jukka Takala, Ilkka Korhonen, Stephan M Jakob

**Affiliations:** 1 Department of Intensive Care Medicine, Bern University Hospital and University of Bern, Freiburgstrasse, CH-3010 Bern, Switzerland; 2 VTT Technical Research Centre of Finland, Tekniikankatu, Tampere, FI-02044 VTT, Finland

## Abstract

**Introduction:**

Sedative and analgesic drugs are frequently used in critically ill patients. Their overuse may prolong mechanical ventilation and length of stay in the intensive care unit. Guidelines recommend use of sedation protocols that include sedation scores and trials of sedation cessation to minimize drug use. We evaluated processed electroencephalography (response and state entropy and bispectral index) as an adjunct to monitoring effects of commonly used sedative and analgesic drugs and intratracheal suctioning.

**Methods:**

Electrodes for monitoring bispectral index and entropy were placed on the foreheads of 44 critically ill patients requiring mechanical ventilation and who previously had no brain dysfunction. Sedation was targeted individually using the Ramsay Sedation Scale, recorded every 2 hours or more frequently. Use of and indications for sedative and analgesic drugs and intratracheal suctioning were recorded manually and using a camera. At the end of the study, processed electroencephalographical and haemodynamic variables collected before and after each drug application and tracheal suctioning were analyzed. Ramsay score was used for comparison with processed electroencephalography when assessed within 15 minutes of an intervention.

**Results:**

The indications for boli of sedative drugs exhibited statistically significant, albeit clinically irrelevant, differences in terms of their association with processed electroencephalographical parameters. Electroencephalographical variables decreased significantly after bolus, but a specific pattern in electroencephalographical variables before drug administration was not identified. The same was true for opiate administration. At both 30 minutes and 2 minutes before intratracheal suctioning, there was no difference in electroencephalographical or clinical signs in patients who had or had not received drugs 10 minutes before suctioning. Among patients who received drugs, electroencephalographical parameters returned to baseline more rapidly. In those cases in which Ramsay score was assessed before the event, processed electroencephalography exhibited high variation.

**Conclusions:**

Unpleasant or painful stimuli and sedative and analgesic drugs are associated with significant changes in processed electroencephalographical parameters. However, clinical indications for drug administration were not reflected by these electroencephalographical parameters, and barely by sedation level before drug administration or tracheal suction. This precludes incorporation of entropy and bispectral index as target variables for sedation and analgesia protocols in critically ill patients.

## Introduction

Pain, physical discomfort and anxiety are common in critically ill patients. The underlying disease, care procedures, prolonged immobility and sleep deprivation all contribute to this [1,2]. Both the stress response and its treatment may have a negative impact on outcome [3-9]. Strategies aiming to reduce the amount of sedatives and analgesics administered may improve outcome and reduce the need for mechanical ventilation [8,10,11]. Accordingly, a reliable, objective assessment of sedation and analgesia during the course of critical illness would be very valuable.

Ideally, sedation in the intensive care unit (ICU) should result in a calm patient who can easily be aroused and has a maintained sleep-wake cycle. Reaching this ideal target is difficult [12], and some patients require deeper levels of sedation, for instance to facilitate circulatory and respiratory support [12]. In addition, patients' requirements for sedative and analgesic drugs vary substantially during the disease process and during therapeutic and supportive interventions.

The clinical assessment of sedation relies on the patient's response to external stimuli. However, the stimulus itself alters the patient's level of sedation. Monitoring electroencephalogram (EEG)-based variables can allow continuous assessment of the level of sedation, and thereby predict the patient's responsiveness. Methods and devices based on processed EEG signals are widely used to monitor the depth of anaesthesia. They have also been advocated for monitoring sedation in intensive care, although the results are controversial. Early observational studies found a good correlation between the Sedation Agitation Scale and bispectral index (BIS) or entropy values [13,14], but other reports could not confirm better performance when compared with standard subjective assessment scores [15-19]. A major drawback of these studies was the fact that the assessment of the sedation score and comparison with the EEG was done in patients who were clinically stable and did not have adjustments to sedation before the assessment. This reflects the difficulties of incorporating processed EEG variables into sedation protocols, because in everyday practice patients need sedation adjustment. The need for these adjustments is usually evaluated by the care teams, with the bedside nurse having a leading role in this assessment because they are with the patient most of the time. The so-called 'gold standard' of sedation can thus be considered to be protocol guided, with goals established by the physician and adjustments made by the bedside nurse.

In clinical routine, many other parameters are used (together with or without a sedation score) to decide whether analgesic or sedative drugs should be administered (including haemodynamic parameters, previous reactions to similar interventions, and sympathetic and parasympathetic reactions). In our experience, these variables do not necessarily correlate with Ramsay Sedation Scale (RSS) score. Reducing the whole sedation process to a single number is not promising; we therefore aimed to describe the indications for drug administration, and monitored patterns in clinical signs and EEG, in order to evaluate whether these patterns can predict the responses in EEG variables. We believe that it is useful to characterize how different interventions and their combinations affect EEG variables in the real-world ICU environment. Studies such as ours can determine the potential of these variables for monitoring various aspects of sedation and analgesia in the context of unpleasant stimuli.

The aim of this observational study was to evaluate different processed EEG parameters as predictors of response to sedative and opiate drugs and intratracheal suctioning, alone or in combination with drugs, during nurse-driven, protocol-guided sedation and analgesia. The interventions were administration of a sedative drug or opiate, clinically indicated endotracheal suctioning, and a combination of both. Specifically, we evaluated whether BIS and state entropy monitoring allow detection of clinically relevant distinctions between light and deep grades of sedation, and help to predict the response to unpleasant care interventions. We hypothesized that there are thresholds beyond which drugs and intratracheal suctioning do not result in significant changes in the respective processed EEG parameters, and that the thresholds for reactions to intratracheal suctioning are modified by prior drug application.

## Materials and methods

The study was approved by the ethics committee of the Canton of Bern, and written informed consent was obtained from the next of kin and, if possible, from the patient after recovery. Inclusion criteria were mechanical ventilation for 48 hours or less and expected need for further ventilation for at least 24 hours. Exclusion criteria were need for muscle relaxation, traumatic brain injury, deep coma due to intoxication or neurological injuries, severe neuropathies or myopathies, and surgery using cardiopulmonary bypass without confirmation of normal neurology before inclusion.

Routine haemodynamic monitoring and treatment were performed according to the decision of the treating physician and standard protocols. In addition, a Datex-Ohmeda S/5 Monitor (Datex-Ohmeda, GE, Helsinki, Finland) was used for measurement and storage (via WinCollect^® ^software [Datex-Ohmeda, GE Healthcare, Helsinki, Finland]) of the following parameters: heart rate, arterial blood pressure (systolic, diastolic and mean), pulse oximetry, end-tidal carbon dioxide tension, and respiratory pressures and volumes. BIS-Index, a processed EEG [20], was recorded via the BIS-Module of the S/5 monitor (XP-Level, smoothing time 15 seconds, using Quattro^® ^Sensor [Datex-Ohmeda, GE Healthcare, Helsinki, Finland]). Entropy is an EEG-derived parameter that uses nonlinear statistics to describe the order of random repetitive signals. The Entropy^® ^Module (Datex-Ohmeda, GE) calculates two indices: the state entropy (SE) and the response entropy (RE). The RE includes additional information about the electromyographic activity (activity higher than 32 Hz) of the face muscles [21]. The SE (range 90 to 0) and the RE (range 100 to 0) are normalized in such a way that the RE becomes equal to the SE when there is no electromyographic activity [22]. Both EEG sensors were attached on the patient's forehead in accordance with the manufacturer's recommendations. BIS and entropy sensors were randomly attached on both sides with the Fz electrode to the upper and lower forehead, respectively.

A simple computer program (annotation board) was developed to help nurses to record the following interventions, defined as events: sedative and analgesic drug bolus, increasing or decreasing continuous sedation and analgesia, intratracheal suctioning, and other potentially painful interventions (for example, chest tube insertion). In addition, the reasons for pharmacological interventions were recorded, as follows: agitation with threat to patient or nurse; agitation; insufficient sedation according to prescription; under-sedation/medical reasons (fighting the ventilator, heart-lung interaction); reduction because of over-sedation level according to prescription; anticipated painful stimulus; pain, as either indicated by the patient or perceived by the nurse subjectively, or based on vegetative signs exhibited by the patient; or opiates to sedate the patient.

A web camera with movement detector was attached above the patient's bed to facilitate the *post hoc *identification of the exact time of the event. Sedative and analgesic drugs were given in accordance with a standard protocol, using sedation goals (RSS score [23]) and regular assessment of sedation and pain at 2-hour intervals or more frequently. Standard doses of fentanyl were 25 to 50 μg, of midazolam were 1 to 2 mg, and of propofol were 10 to 20 mg. If more than six boli were needed in a 4-hour period, continuous infusion of the respective drug was started. A daily sedation stop was conducted unless the attending physician explicitly ordered otherwise. Reduction in continuous medication at 2-hour intervals was encouraged. Screening for delirium was not routinely conducted at that time, and so only overt delirium was detected, but no patient in this study received an antipsychotic drug (haloperidol). All medications were prescribed by the treating physician and applied by the bedside nurse, both of whom were blinded to the EEG parameters. The bedside nurse was free to administer drugs within the prescribed limits before a painful stimulus, such as intratracheal suctioning. The main reasons for administering drugs were anticipation of arterial oxygen desaturation, pain, or heart-lung interaction. The EEG-derived variables (BIS-Index, RE, SE, 60 sec mean values) and physiological parameters were recorded continuously, and were analyzed at 30 and 2 minutes before the event (time points -30 and -2), and at 2, 5 and 10 minutes after the event (time points +2, +5, +10).

The study was performed for 24 hours or until extubation, if earlier. Afterward, the camera recordings were analyzed and any missing annotations were completed. For all recorded haemodynamic, respiratory and neurological parameters, mean values over 60 seconds were calculated at 30 and 2 minutes before the intervention (stimulus or drug) and at 2, 5 and 10 minutes after the intervention. Because the events were not planned, RSS score were not available at all time points of EEG processing. Only RSS scores assessed shortly before the event (< 15 minutes) were used for further analysis.

Because BIS-Index and Entropy are ordinal scale based, nonparametric tests for independent or repeated measures were used. Dunn's method was used for multiple pair-wise comparisons. Comparisons of continuous variables were conducted after running a normality test (Kolmogorov-Smirnov), with the appropriate parametric or nonparametric test, as indicated in the tables. Receiver operating characteristic (ROC) curves were used to define best cut-off values for definition of responders to medication (decrease of the BIS-Index or SE/RE), with the increase in the processed EEG variable between the time points -30 minutes and -2 minutes as test variable. Statistical analyses were conducted using the SigmaStat for Windows Version 3.1 software package (Systat Software Inc., Point Richmond, CA, USA). A *P *value under 0.05 was considered statistically significant. ROC curves were constructed with the SigmaPlot for Windows Version 10.0 software package (Systat Software Inc.).

## Results

**Figure 1:**
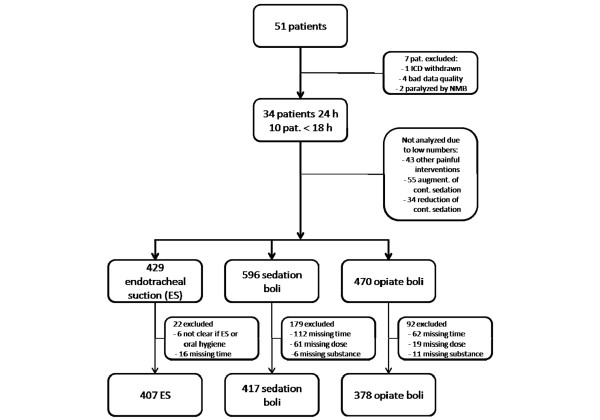
Diagram showing the numbers of patients and events ultimately used for analysis. NMB, neuromuscular blockade. ICD: informed consent (document).

Fifty-one patients were included in the study (Table 1). Seven patients were excluded after the study because of withdrawal of informed consent (*n* = 1), insufficient EEG quality (*n* = 4) and intermittent, unanticipated use of muscle relaxants (*n* = 2). The median recording time was 23 hours (from 12:30 to 27:10 hours). Altogether, 1,722 events were identified, of which 388 (23%) had to be excluded from analysis, mostly because of missing annotations and failure to classify the event clearly despite using video recordings. For in-depth analyses, we considered the 407 endotracheal suctioning episodes, the 417 sedation boli and the 378 opiate boli (Figure 1). RSS score assessments in close proximity to time point -2 (2 minutes before an event) were available for 695 events. Events with low incidences (< 5% of total) were excluded from detailed analysis (Figure 1).

**Table 1:** Patient characteristics, and sedative and analgesic drugs used

Characteristic	Value
Age (years median [range])	66 (38 to 83)

Diagnosis (*n*)	

ACS/circulatory failure	12
Respiratory failure (pneumonia, COPD)	11
Sepsis (other than pneumonia)	9
Trauma/major emergency surgery	4
Other	8
Sedation (*n*)	

Midazolam	24
Propofol	17
Opiate only	3
Opiate (*n*)	

Fentanyl	41
Sedation only	3

A total of 44 patients were included in the study. ACS, acute coronary syndrome; COPD, chronic obstructive pulmonary disease.

### Relationship between EEG-derived variables and clinical sedation level

**Figure 2:**
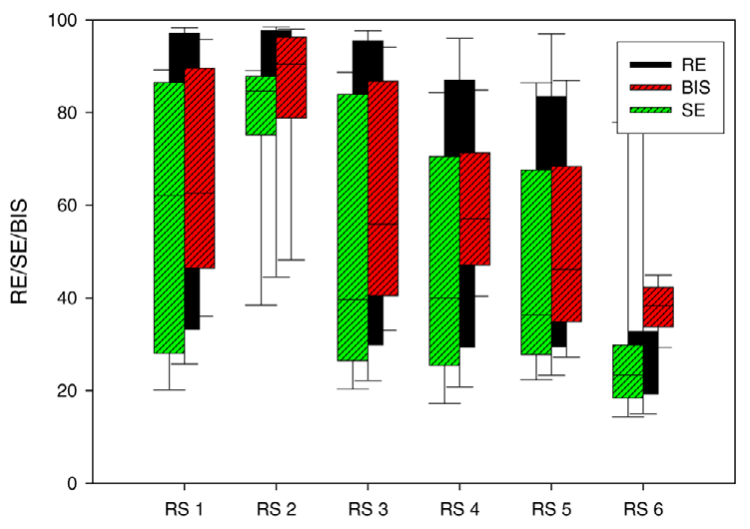
Response entropy/BIS-Index/state entropy at different Ramsay Sedation Scale scores. The 1,932 data points (about 660 events) are at -2 minutes (2 minutes before an event). Boxes show median, 25th and 75th percentiles; whiskers indicate the 10th and 90th percentiles. RE, response entropy; RS, Ramsay Sedation Scale; SE, state entropy.

All EEG-derived variables correlated with the clinical level of sedation (*r *= -0.372 for RE [*n* = 679]; *r *= -0.360 for SE [*n* = 679]; and *r *= -0.426 for BIS [*n* = 604]; all *P *< 0.001), but the overlap between the clinical sedation levels was wide (Figure 2). None of the processed EEG variables was able to discriminate between light to moderate sedation (RSS scores 1 to 4) and deep sedation (RSS scores 5 to 6; Figure 2 and Table 2). Although the differences were statistically significant, the first quartiles of the light to moderately sedated patients' EEG parameters were below the third quartiles of the other groups, indicating clinically important overlap. Analysis of subgroups of the events, namely sedation bolus, opiate bolus and endotracheal suction, did not reveal any groups in which the processed EEG performed better (see Additional data file 1).

**Table 2:** Processed EEG parameters at 2 minutes before the event, separated by patients with light versus deep sedation

	RE	SE	BIS	P values
All events				

RSS score 1 to 4 (*n* = 539)	79 (35 to 97)	61 (30 to 86)	66 (48 to 89)	All *P *< 0.001 (Mann-Whitney)
RSS score > 4 (*n* = 160)	34 (26 to 58)	31 (24 to 48)	41 (34 to 58)	

Sedation boli				

RSS score 1 to 4 (*n* = 192)	85 (39 to 97)	73 (34 to 86)	67 (49 to 91)	All *P *< 0.001 (Mann-Whitney)
RSS score > 4 (*n* = 60)	33 (26 to 48)	31 (33 to 51)	40 (33 to 51)	

Opiate boli				

RSS score 1 to 4 (*n* = 179)	56 (30 to 96)	43 (26 to 85)	59 (46 to 83)	All *P *< 0.001 (Mann-Whitney)
RSS score > 4 (*n* = 60)	31 (23 to 41)	29 (31 to 44)	40 (31 to 44)	

ETS				

RSS score 1 to 4 (*n* = 168)	89 (45 to 97)	75 (36 to 86)	74 (50 to 91)	RE: *P *= 0.012
RSS score > 4 (*n* = 40)	50 (30 to 92)	44 (27 to 80)	60 (44 to 80)	SE: *P *= 0.018 BIS: *P *= 0.058

RSS score 1 to 4 indicates light sedation, and RSS score > 4 indicates deep sedation. Values are expressed as median (interquartile range). BIS, bispectral index; EEG, electroencephalogram; ETS, endotracheal suctioning; RE, response entropy; RSS, Ramsay Sedation Scale; SE, state entropy.

Evaluation of the individual correlation coefficients of 22 patients in whom at least eight simultaneous measurements of RSS score and processed EEG could be recorded did not reveal any patients who had high coefficients (data not shown). Therefore, the existence of some individuals with good correlations of EEG parameters and RSS score appears unlikely.

### Reasons for increasing the level of sedation and analgesia and the effect on EEG-derived variables

We recorded 417 events for which sedation boli were administered. The most common indication was agitation (*n* = 149), followed by anticipation of unpleasant intervention (*n* = 116) and fighting the ventilator/heart-lung interactions/adverse circulatory effects (*n* = 86). No specific reason was recorded for 57 events. For the agitation indication, all EEG-derived variables 2 minutes before drug administration, sedation levels (RSS score) and the blood pressure differed in comparison with the other indications (*P *< 0.001; Table 3). The EEG-derived variables indicated deepest sedation in patients receiving additional sedation due to fighting the ventilator or heart-lung interactions (medical reasons in Table 3).

**Table 3:** Processed EEG and physiological variables and RSS score 2 minutes before sedation boli

Variable	Indication					*P*	Test
			
	Agitation with threat	Agitation	Medical reasons	Anticipated nursing procedure	No annotation		
*n*	20	129	86	116	57		
Response entropy	91 (61 to 97)	91 (44 to 98)	35 (22 to 65)	48 (28 to 96)	65 (32 to 96)	< 0.001	Kruskal-Wallis
State entropy	79 (54 to 86)	79 (40 to 88)	32 (21 to 56)	43 (26 to 74)	59 (29 to 85)	< 0.001	Kruskal-Wallis
BIS-Index	81 (71 to 91)	76 (54 to 91)	48 (40 to 62)	57 (47 to 72)	72 (46 to 92)	< 0.001	Kruskal-Wallis
RSS score	1 (1 to 2)	2 (1 to 4 to)	4 (3 to 5)	4 (2 to 4)	4 (2 to 4)	< 0.001	Kruskal-Wallis
Heart rate (beats/minute)	83 (69 to 104)	90 (73 to 104)	96 (90 to 107)	92 (72 to 100)	96 (85 to 107)	0.011	Kruskal-Wallis
etCO_2 _(mmHg)	45 (37 to 62)	42 (34 to 55)	47 (35 to 57)	41 (36 to 53)	34 (33 to 50)	0.043	Kruskal-Wallis
FiO_2 _(%)	48 (40 to 62)	47 (39 to 58)	50 (39 to 61)	48 (40 to 59)	39 (38 to 54)	0.062	Kruskal-Wallis
Respiratory rate (breaths/minute)	19.0 (16.3 to 19.8)	15.5 (12.0 to 20.0)	17.8 (12.0 to 20.0)	14.3 (11.8 to 18.3)	17.7 (11.7 to 20.4)	0.025	Kruskal-Wallis
SpO_2 _(%)	95 (92 to 98)	96 (92 to 98)	97 (95 to 98)	95 (93 to 98)	96 (95 to 98)	0.116	Kruskal-Wallis
SBP (mmHg)	115 ± 22	113 ± 21	96 ± 28	110 ± 26	113 ± 29	< 0.001	RM-ANOVA
MBP (mmHg)	79 (71 to 82)	70 (63 to 79)	63 (57 to 69)	69 (59 to 79)	70 (63 to 84)	< 0.001	Kruskal-Wallis
DBP (mmHg)	60 (55 to 64)	50 (44 to 59)	46 (39 to 52)	50 (41 to 58)	52 (45 to 60)	< 0.001	Kruskal-Wallis

The sedation boli were given for various indications, according to the nurses' notes. Values are expressed as median (interquartile range) or as mean ± standard deviation. BIS, bispectral index; SBP, MBP, DBP, systolic, mean, diastolic blood pressure; EEG, electroencephlogram; etCO_2_, end-tidal barbon dioxide; FiO2, fractional inspired oxygen; RM-ANOVA, repeated measures analysis of variance; RSS, Ramsay Sedation Scale; SpO_2_, pulse oximetry.

The indication for the administration of the 378 opiate boli was most often anticipated pain during planned nursing (*n* = 73), followed by agitation as a sign of pain (rated subjective by nurse; *n* = 60) and agitation as a sign of pain (rated objective as indicated by clinical signs; *n* = 56). The patient asked for pain relief in 39 cases. Anticipation of pain during surgical tasks (for example, wound dressing) was rare (*n* = 20), and administration of opiate boli to reduce sedation was the exception (*n* = 9). No indication was noted 60 times, and various indications were given in 49 events. Deepest processed EEG values were registered with the anticipated pain indications, in the sedation-sparing indication and in agitated patients with objective signs of pain (Table 4).

**Table 4:** Processed EEG and physiological variables, and RSS score 2 minutes before opiate boli

Variable	Indication								*P*
		
	0: left blank/missing	1: patient asks	2: agitation, nurse thinks of pain	3: agitation, objective signs of pain	4: anticipated pain (surgical)	5: anticipated pain (nursing)	6: to reduce sedatives	7: comment	
*N*	60	39	60	56	16	73	9	49	
Response entropy	73 (27 to 97)	94 (35 to 98	53 (30 to 96	36 (25 to 63	28 (19 to 34	32 (25 to 81	17 (11 to 25	81 (33 to 96	< 0.001
State entropy	61 (25 to 87)	81 (25 to 84)	40 (24 to 87)	30 (22 to 56)	24 (18 to 29)	28 (23 to 71)	15 (10 to 19)	65 (31 to 83)	< 0.001
BIS-Index	66 (45 to 92)	77 (62 to 93)	62 (43 to 82)	51 (40 to 59)	49 (40 to 52)	53 (42 to 73)	46 (40 to 56)	54 (32 to 73)	< 0.001
RSS score	4 (2 to 5)	2 (2 to 3)	3 (1 to 4)	3 (2 to 4)	5 (3 to 5)	4 (3 to 4)	4 (2 to 4)	4 (2 to 5)	< 0.001
Heart rate (beats/minute)	92 (81 to 102)	90 (72 to 101)	96 (90 to 107)	100 (94 to 109)	97 (90 to 110)	93 (85 to 100)	95 (93 to 100)	86 (74 to 95)	< 0.001
etCO_2 _(mmHg)	36 (33 to 55)	34 (32 to 46)	41 (34 to 50)	48 (36 to 55)	43 (41 to 49)	41 (35 to 48)	54 (47 to 56)	34 (27 to 45)	< 0.001
FiO_2 _(%)	41 (38 to 59)	39 (38 to 53)	44 (39 to 53)	53 (41 to 57)	46 (45 to 52)	45 (39 to 53)	58 (51 to 60)	39 (34 to 48)	< 0.001
Respiratory rate (breaths/minute)	14.0 (11.8 to 19.9)	12.8 (10.0 to 19.0)	16.8 (12.2 to 20.9)	19.8 (16.6 to 22.0)	16.7 (13.6 to 20.0)	16.5 (12.0 to 19.8)	18.3 (13.5 to 19.0)	13.6 (12.0 to 18.0)	< 0.001
SpO_2 _(%)	96 (93 to 98)	96 (93 to 97)	95 (94 to 98)	95 (93 to 97)	98 (96 to 98)	96 (94 to 98)	96 (94 to 97)	95 (94 to 97)	0.053
SBP (mmHg)	111 (99 to 126)	119 (104 to 146)	113 (102 to 133)	101 (91 to 113)	94 (76 to 102)	109 (96 to 120)	65 (53 to 116)	105 (99 to 116)	< 0.001
MBP (mmHg)	69 (61 to 78)	77 (71 to 83)	70 (66 to 81)	66 (62 to 73)	56 (48 to 68)	70 (62 to 75)	51 (44 to 79)	67 (62 to 74)	< 0.001
DBP (mmHg)	51 (42 to 59)	55 (48 to 59)	53 (47 to 59)	50 (45 to 54)	38 (28 to 44)	51 (43 to 58)	34 (34 to 43)	50 (47 to 55)	< 0.001

The opiate boli were given for various indications, according to the nurses' notes. Values are expressed as median (interquartile range). All P values were calculated using the Kruskal-Wallis test. BIS, bispectral index; DBP, diastolic blood pressure; EEG, electroencephlogram; etCO_2_, end-tidal barbon dioxide; FiO2, fractional inspired oxygen; MBP, mean blood pressure; RSS, Ramsay Sedation Scale; SBP, systolic blood pressure; SpO_2_, pulse oximetry.

**Figure 3:**
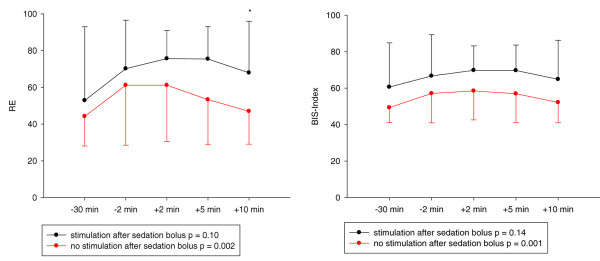
Time courses of response entropy and BIS-Index after sedation bolus. Black lines and red lines indicate stimulus (endotracheal suction) and no stimulus within 10 minutes of the sedation bolus. Dots are medians, and the error bars indicate the 25th and 75th percentiles. The asterisk denotes a significant difference (*P *< 0.05) between the groups at 10 minutes. RE, response entropy.

Patients responded to a sedation bolus with a significant decrease in all processed EEG values. When endotracheal suctioning was performed within 10 minutes after a sedation bolus, the effect on processed EEG variables was attenuated (Figure 3).

Neither increase in RE nor that in BIS from time point -30 minutes to -2 minutes was a good predictor of a strong response 10 minutes after the sedation bolus. ROC curves with a sedation response (defined as a decrease in the processed EEG variable of at least 15%, 20% and 25% after sedation bolus) are shown in the Additional data file 2. The areas under the ROC curve were between 0.70 and 0.75 for RE and between 0.74 and 0.80 for BIS.

### Response to unpleasant stimuli

**Figure 4:**
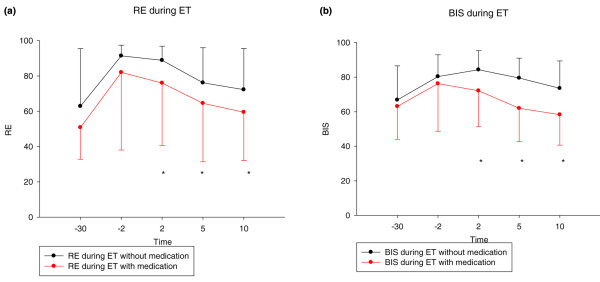
Time course of RE and BIS-Index during endotracheal suctioning episodes. **(a)** Time course of RE during the endotracheal suctioning episodes (ET), without (black) and with (red) medication before ET. Asterisks denote significant differences (*P *< 0.05) between the groups at these time points. **(b) **Time course of BIS-Index during the ETs, without (black) and with (red) medication before ET. Asterisks denote significant differences (*P *< 0.05) between the groups at these time points. RE, response entropy.

In 103 instances patients received sedative and/or analgesic drugs before intratracheal suctioning, whereas in 282 instances patients did not. EEG-derived variables exhibited no difference between the groups at -30 minutes or -2 minutes before the unpleasant stimulus (Figure 4 and Table 5). There were also no significant or clinically relevant differences in physiological parameters, such as heart rate, blood pressure and respiration 2 minutes before endotracheal suctioning (Table 5). Patients who never received medication before suctioning, or who received medication less than 50% of the time, did not differ with respect to age, Simplified Acute Physiology Score or length of stay in the ICU from patients who always or almost always received medication before suctioning (Table 4).

**Table 5:** Processed EEG and physiological parameters 2 minutes before endotracheal suctioning, with and without medication given up to 10 minutes before endotracheal suctioning

Variable	Without medication	With medication	*P*	Test
*N*	282	103		
Response entropy	91 (47 to 97)	82 (39 to 97)	0.111	Mann-Whitney
State entropy	78 (42 to 87)	66 (31 to 85)	0.101	Mann-Whitney
BIS-Index	80 (57 to 93)	76 (49 to 91)	0.078	Mann-Whitney
Heart rate (beats/minute)	83 (70 to 99)	91 73–100)	0.09	Mann-Whitney
etCO_2 _(mmHg)	37 (33.44)	41 (34 to 53)	< 0.01	Mann-Whitney
FiO_2 _(%)	40 (38 to 49)	47 (38 to 58)	0.02	Mann-Whitney
Respiratory rate (breaths/minute)	14.0 (11.0 to 18.0)	14.5 (11.6 to 19.0)	0.43	Mann-Whitney
SpO_2 _(%)	95 (93 to 79)	95 (93 to 97)	0.92	Mann-Whitney
SBP (mmHg)	116 (103 to 138)	117 (103 to 134)	0.83	Mann-Whitney
MBP (mmHg)	74 (67 to 83)	75 (65 to 81)	0.45	Mann-Whitney
DBP (mmHg)	55 (49 to 61)	53 (44 to 60)	0.19	Mann-Whitney
RSS score	2 (2 to 4; n = 147)	3 (2 to 4; n = 61)	0.25	Mann-Whitney
Age (years)	66.6 ± 12.3	60.0 ± 10.9	0.11	*t*-test
SAPS II score	47.7 ± 19.2	42.2 ± 16.0	0.40	*t*-test
LOS (minutes)	10,750 (5,230 to 195,90)	10,305 (6,600 to 12,210)	0.99	Mann-Whitney

Because of the unbalanced numbers of events, age, SAPS and LOS were divided into two groups: endotracheal suctioning without medication or with medication less than 50% of the time, and endotracheal suctioning with medication more than 50% of the time or always with medication. Values are expressed as median (interquartile range) or as mean ± standard deviation. BIS, bispectral index; DBP, diastolic blood pressure; EEG, electroencephlogram; etCO_2_, end-tidal barbon dioxide; FiO2, fractional inspired oxygen; LOS, length of stay; MBP, mean blood pressure; RSS, Ramsay Sedation Scale; SAPS, Simplified Acute Physiology Score; SBP, systolic blood pressure; SpO_2_, pulse oximetry.

**Figure 5:**
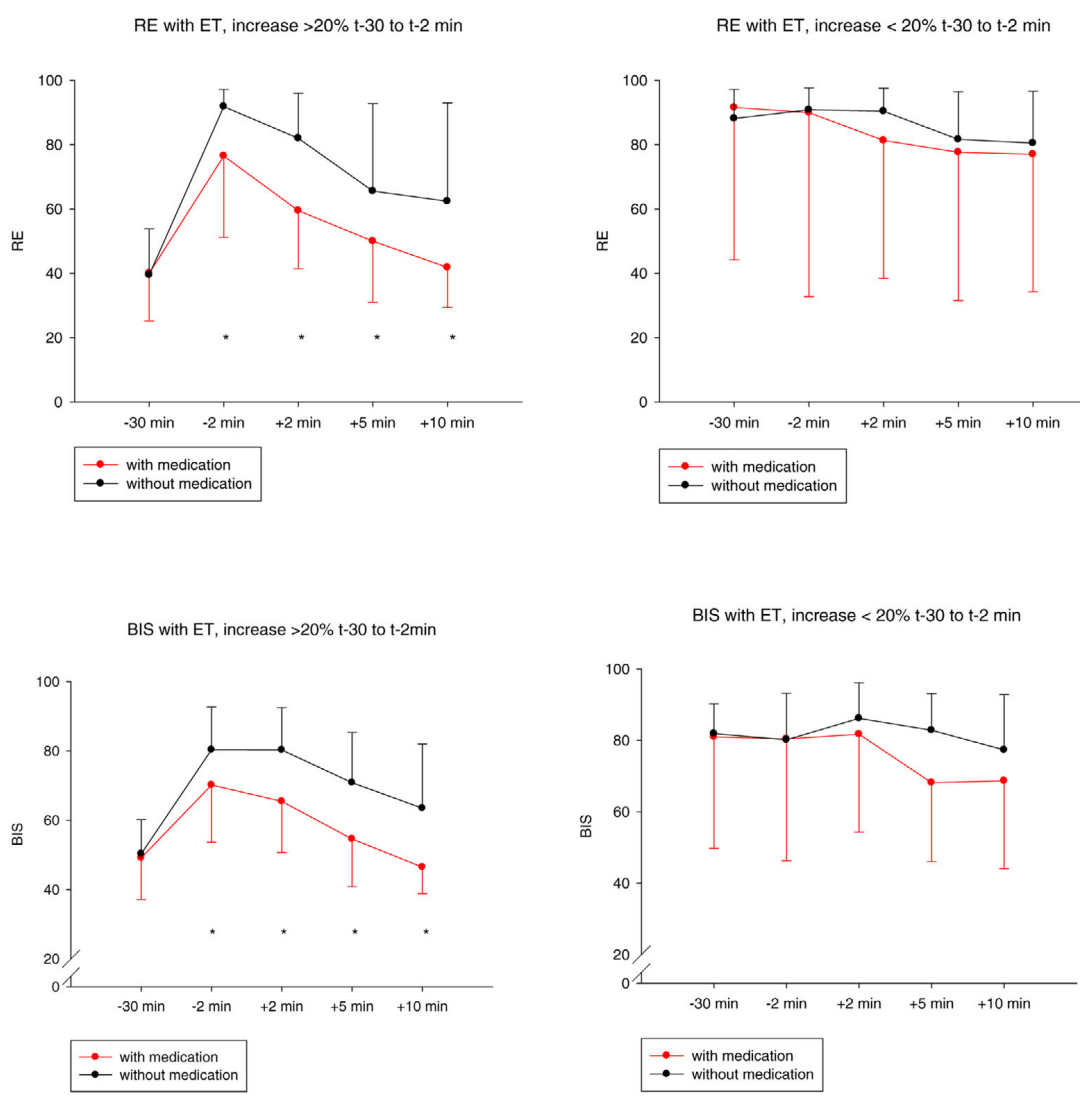
Time course of EEG variables during endotracheal suctioning episodes. Findings are stratified by the increase in EEG parameters (> 20% versus < 20%) before endotracheal suctioning episodes (ET), with and without medication before ET. Asterisks denote significant differences between the groups at these time points (*P *< 0.05). EEG, electroencephalogram.

Patients with a 20% or greater increase in the processed EEG variables between -30 minutes and -2 minutes reached their baseline level faster if they had medication before suctioning, whereas patients with an increase of less than 20% did not show any difference (see Figure 5).

As with sedation bolus alone, neither increase in RE nor that in BIS from time point -30 minutes to -2 minutes was a good predictor of a response 10 minutes after the endotracheal suction with pre-medication. ROC curves with different sedation responses are shown in the Additional data file 2. The areas under the ROC curve were between 0.77 and 0.83 for RE and between 0.78 and 0.80 for BIS.

Because several patients had sepsis, delirium or septic encephalopathy was likely. We therefore divided the patients into a nonseptic and a septic group, which revealed that processed EEG readings are lower in septic patients in some occasional clinical scenarios, whereas the RS at -2 minutes was the same throughout (see Additional data file 3).

## Discussion

In the present observational study, which included additional verification through a camera, we have created an unprecedented and large database of sedation events and unpleasant stimuli in real-life patients. Furthermore, the decision not to allocate study personnel for bedside annotations has minimized effects of the study set-up (per se) on the nurses' decisions. These data probably represent the largest study of EEG-derived parameters in an ICU population outside the setting of a controlled study. Sedation is a multidimensional concept, encompassing consciousness, amnesia, arousal, analgesia and other parameters, and is difficult to represent using a single scale. Failure in clinical practice to capture all aspects of sedation and analgesia within a sedation scale is an unsolved problem, as corroborated by the present study. The RSS score [23], for example, is unbalanced and favours sedation aspects; the Richmond Agitation and Sedation Score [17] and the Sedation-Agitation Score [24] are more balanced, but they lack the means to detect delirium or pain as a cause of agitation. No score can predict arousal in ICU patients. It is possible that failure to monitor all aspects of sedation in the present study accounts for the proportion of missing details regarding reasons for drug administration, and especially the large proportion of additional comments given as reasons for opioid administration. For example, nurses gave medication for the indication 'heart-lung interaction/fighting the ventilator' based on their observations of patient response during previous interventions despite deep sedation. The relatively high frequency of opiate and sedative administration despite deep sedation in anticipation of interventions and for diverse medical reasons (for instance, fighting ventilator and heart-lung interactions) represents further evidence of the problems associated with current sedation scores.

Taking a broader view, this might be the reason why, even now, not all sedated patients in the ICU are monitored and guided using a sedation score, as was recently confirmed by Payen and coworkers [25]. As pointed out by Carlon and Combs [26], 'If you cannot measure it, you cannot improve it.' Unfortunately, with the use of our sedation protocol, EEG-derived parameters do not add helpful information in terms of guiding the administration of sedatives or analgesics for the most frequently occurring indication, namely agitation. With respect to the second most used indication for sedatives and opiates, namely anticipated pain during nursing or endotracheal suctioning, neither EEG-derived parameters, an increase in these parameters before suctioning, nor RSS score can identify which patients might profit from prophylactic drug administration. Because patients receiving prophylactic drugs have statistically significant but clinically only slightly worse lung function parameters, and because the EEG-derived parameters reach their baseline levels faster after drug administration, we could speculate that nurses have previously noted a clinical benefit for some patients and have used prophylactic drugs in those patients who benefit most. However, these patients cannot be identified using processed EEG parameters, and it is likely that prophylactic use of drugs is an aspect of individual nurse behavior and has no rationale. For the third most often used indication for sedation, namely fighting the ventilator/heart-lung interaction, we also identified a correlation between lower EEG-derived parameters and lower sedation levels, but these parameters are unhelpful in deciding whether to extend the sedation protocol, because neither the clinical score nor the EEG parameters identify the indication and precise time point when the drug should be given.

Regarding endotracheal suctioning, it was surprising that patients who received drugs before the unpleasant stimulation had a lower RSS score than those who did not receive the medication, reflected in higher processed EEG variables, although both of these associations were not statistically significant. They also had a slightly lower EEG reading at +2 minutes as compared with -2 minutes, which mirrors the effect of the sedation bolus. It could be argued that patients receiving medication before the event have a lower EEG reading, and that this is certainly due to the medication. However, if the time between -30 minutes and -2 minutes is taken into account, then the principal component of the rise in RE/SE/BIS lies before the intervention, and so the need for suctioning elicits more arousal than the suctioning itself. In turn, the use of medication to attenuate the response to suctioning is less responsible for the return of RE/SE/BIS to baseline than the cessation of suctioning itself. So, it may be debated whether prophylactic use of drugs before suctioning should be limited to special groups of patients who cannot tolerate suctioning, such as those with heart-lung interactions or high intracranial pressure. This might reduce the total amount of drugs given to the patients and therefore decrease the total ventilation time, as demonstrated by various investigators [8,11].

The wide overlap of the parameters RE/SE and BIS-Index precludes the use of these variables as crude parameters for discrimination of light/moderate/deep sedation in our patient population. After initial enthusiasm over the use of the BIS-Index as a parameter of sedation in ICU patients [13,14], confirmatory studies have found the wide overlap of the BIS-Index to be problematic, although the BIS-XP technology can identify and better integrate artifactual EEGs in ICU patients [15-17]. Still less has been published with regard to Entropy^® ^in ICU patients, but its use in this patient population was also discouraged in a recent report [18].

The strength of studying real patients and patient-nurse interactions is also a potential weakness of this study. Adherence to the sedation protocol was not stringent, and a significant portion of drug administration was recorded only in the nurses' notes and not in the annotation board, especially with regard to analgesic drug administration.

The lack of RSS scores collected concomitantly with processed EEG variables at all recording time points is also a limitation of the study. Concomitant assessment of the clinical degree of sedation and EEG parameters would have allowed the relationship between the two to be addressed in greater detail. Our study design did not allow this because the 'event' could not be precisely anticipated. In addition, evaluating the RSS score changes the EEG *per se*. There is a wide variation in methods of timing and interpretation of EEG in conjunction with clinical sedation assessment in the literature. Some authors used a steady state at least 15 minutes from the event, and manually averaged EEG values were only used when there was a stable period [13]; others collected the EEG values during the assessment [14,17] or before assessment [15,18], and still others took values only if the patient was not arousable (at RSS score 6) [16].

## Conclusion

Unpleasant or painful stimuli and use of sedative and analgesic drugs are associated with significant changes in processed EEG parameters. However, clinical indications for drug administration were not reflected by these EEG parameters, and were barely reflected by sedation level before drug administration or tracheal suction. The use of a sedation score, as recommended in a recent guideline [12], is far from perfect, and the need for sedation in special circumstances such as heart-lung interactions or when patients fight the ventilator is not reflected in sedation scores. Given that the poor quality of sedation and difficulties in reaching and maintaining sedation targets cannot be resolved with currently available processed EEG methods or scores, how to achieve optimal sedation remains a major problem in the ICU.

## Key messages

• Sedation scores do not predict arousal and may not be helpful in guiding sedation in some clinical settings.

• BIS-Index and Entropy do not add information which can be used to guide sedation in the general ICU population.

## Abbreviations

EEG: electroencephalogram; ICU: intensive care unit; RE: response entropy; ROC: receiver operating characteristic; RSS: Ramsay Sedation Scale; SE: state entropy.

## Competing interests

The study was funded by an unrestricted grant from Instrumentarium/Datex-Ohmeda, now GE Healthcare, Helsinki, Finland. The study design was approved, but not influenced, by GE Healthcare. Instrumentarium/Datex-Ohmeda was not involved in any way in collection, analysis and interpretation of data, in writing of the manuscript, or in the decision to submit this manuscript.

In relation to MH, CB, CS, JT and SMJ, the Department of Intensive Care Medicine has received research funding from GE Healthcare to carry out research projects related to depth of anaesthesia monitoring. A part of the work reported here resulted from these projects.

In relation to HY and IK, the VTT Technical Research Centre of Finland have received funding from GE Healthcare to carry out research projects related to depth of anaesthesia monitoring. Both authors have been working on these research projects, and part of the work reported here resulted from these projects.

## Authors' contributions

MH conceived and designed the study, contributed to acquisition, analysis and interpretation of data, performed the statistical analysis, and drafted the manuscript. HY made substantial contributions to data acquisition and interpretation. CB and CS planned the study, and collected and analyzed the data. This manuscript represents their thesis for Medical Degrees at the University of Bern. JT contributed to study design, data interpretation and drafting of the manuscript. IK contributed to data analysis and revised the manuscript. SJ conceived of the study, and contributed substantially to all parts of the study and manuscript preparation. All authors gave final approval of the version to be published.
